# The role of RANBP1 in regulating MiRNA expression and apoptosis in breast cancer cells

**DOI:** 10.1007/s13258-025-01664-5

**Published:** 2025-08-21

**Authors:** Pengxia Song, Lin Zhang, Huiping Qiu, Xingchen Zhou, Yanhui Cao, Jie Liu, Xin Zheng, Yubin Liu, Shuihong Yao

**Affiliations:** 1 Medical School, Quzhou College of Technology, No.18, Jiangyuan Road, Quzhou, 324000 Zhejiang Province China; 2 Division of (Bio) Pharmaceutics, Institute of Zhejiang University-Quzhou, 99 Zheda Road, Quzhou, 324000 Zhejiang Province China; 3 Quzhou People’s Hospital, The Quzhou Affiliated Hospital of Wenzhou Medical University, Quzhou, 324000 China

**Keywords:** Breast cancer, sh-RANBP1, Apoptosis, miRNA, mRNA

## Abstract

**Background:**

Breast cancer poses a huge health threat in China. Emerging evidence has indicated that RANBP1 is associated with poor prognosis of breast cancer and has been shown to influence miRNA expression in colorectal cancer. But its role in breast cancer remains unclear.

**Objective:**

The purpose of this research is to construct the sh-RANBP1 cell line derived from a human breast cancer cell line in order to investigate the impact of low RANBP1 expression on the expression network of breast cancer-associated miRNAs and mRNAs.

**Methods:**

We constructed the sh-RANBP1 cell line from the human breast cancer cell line MDA-MB-231 and analyzed the significant differential expression between the two groups using miRNA sequencing and mRNA sequencing of both the sh-RANBP1 group and the control group. The differential miRNA-mRNA regulatory network for the two groups was established by intersecting target predictions with the differential expression data. The results were further verified using Western blotting (WB), quantitative PCR (qRT-PCR), and flow cytometry.

**Results:**

In this study, we compared the expression levels of miRNA and mRNA between the sh-RANBP1 group and the control group. Our findings indicate that sh-RANBP1 influences miRNA expression, revealing significant differences based on the interaction network of miRNA and mRNA. The genes involved are associated with pathways such as apoptosis. Subsequent WB and qRT-PCR results further validated our findings. Finally, flow cytometry confirmed an increased proportion of apoptosis in the sh-RANBP1 interference group.

**Conclusion:**

In summary, sh-RANBP1 affects miRNA expression in breast cancer cells, then regulates the expression of mRNA, and ultimately increases the proportion of apoptosis in breast cancer cells.

**Supplementary Information:**

The online version contains supplementary material available at 10.1007/s13258-025-01664-5.

## Introduction

In China, breast cancer has emerged as the most common form of cancer affecting women, and its incidence rates have experienced a substantial increase (Fan et al., 2014). While the age-standardized rate of breast cancer incidence in China is less than that observed in numerous developed nations, it is rising swiftly, especially in urban regions (Lei et al., 2021). In 2015, the incidence of breast cancer was projected to exceed 200,000 cases, with estimates suggesting it could approach 230,000 by 2025 (Wu et al., 2024). The mean age at diagnosis in China is notably younger, typically ranging from 45 to 55 years, compared to older ages in Western countries (Fan et al., 2014). An earlier onset is linked to a greater percentage of premenopausal instances, around 62.9% in China, which is markedly higher compared to the United States and the European Union (Phillips et al., 2009). Although the incidence is increasing, the death rate from breast cancer in China continues to be disturbingly high, particularly in rural regions where healthcare access is restricted (Al et al., 2024). The mortality-to-incidence ratio (M/I) for breast cancer in China is higher than that in many developed nations, indicating that while more women are diagnosed, fewer are surviving the disease (Lei et al., 2021). This disparity is exacerbated by socioeconomic factors, as patients from rural areas often present with more advanced stages of cancer at diagnosis, leading to poorer outcomes (Bradley et al., 2001). Studying the causes and molecular mechanisms of breast cancer presents several challenges due to the disease’s complexity and heterogeneity (Rivenbark et al., 2013). Breast cancer should be viewed not as a singular illness but as a group of subtypes, each exhibiting unique biological traits, genetic alterations, and treatment responses (Yersal and Barutca 2014). This heterogeneity complicates the identification of universal causes and mechanisms that apply across all cases (Kalinowski et al., 2019).

RANBP1, a known cofactor of the RAN protein, plays a role in mitosis and the cell cycle, and has been implicated in cancer malignancy (Kahm et al., 2023). Recent studies have indicated a significant association between RANBP1 and cancer stem cells (CSCs), as well as glioma stem cells (GSCs), suggesting its involvement in the regulation of cancer malignancy through various mechanisms, including the modulation of microRNAs that are crucial for CSCs maintenance (Zheng et al., 2022). In the realm of breast cancer, RANBP1 has been identified as an important marker in survival studies. Patients showing higher levels of RANBP1 expression experienced significantly lower survival rates, emphasizing its potential role as a target for therapy in cancer management (Ochoa et al., 2020). The study posits that RANBP1 could serve both as a therapeutic target and as a novel marker for breast cancer, with its significance anticipated to increase as further research is conducted (Kirmizis et al., 2003). Furthermore, the study emphasizes that RANBP1 regulates the expression of specific microRNAs (miRNAs) that may influence cancer cell behavior, including their metastatic potential (Fu et al., 2024). Notably, RANBP1-mediated regulation of miRNAs may affect breast cancer metastasis through modulation of apoptosis-related genes. Apoptosis resistance is a hallmark of metastatic cancer cells, enabling survival during detachment, invasion, and colonization of distant organs (Rohini et al. 2018). This regulatory role suggests that RANBP1 could potentially contribute to the metastatic process of breast cancer, however, the precise mechanisms and pathways involved warrant further investigation.

Small non-coding RNA molecules known as miRNAs play a crucial role in the regulation of gene expression and have been associated with the development of breast cancer (Loh et al., 2019). In addition to their role in the onset of cancer, miRNAs also play a part in the advancement and spread of breast cancer (Fridrichova and Zmetakova 2019). Alterations in miRNA regulation can increase the ability of cancer cells to migrate and invade, thus promoting metastasis (Zhang et al., 2014). For instance, miR-205 has been shown to be downregulated in breast cancer, with its low expression correlating with advanced tumor stages and poor prognosis (Xiao et al., 2019). Restoring the expression of miR-205 has been shown to suppress cell growth and enhance apoptosis, highlighting its potential as a target for therapy (Vosgha et al., 2014). Moreover, comprehending the biology of breast cancer greatly relies on the regulatory network that includes miRNAs, long non-coding RNAs (lncRNAs), and messenger RNAs (mRNAs) (Crudele et al., 2020). This network involving lncRNA, miRNA, and mRNA has the potential to affect multiple cellular functions, such as the regulation of the cell cycle, apoptosis, and therapeutic responses (Wang et al., 2020). The interplay between lncRNAs and miRNAs can influence the expression of target genes that are associated with the progression of breast cancer, which offers valuable information for potential therapeutic approaches (Wang et al., 2019).

This study seeks to clarify the role of RANBP1 in the progression of breast cancer, with a particular emphasis on how it affects miRNA expression and subsequent cellular activities. By analyzing the regulatory network between RANBP1 and miRNA, along with its functional implications in breast cancer cells, we aim to offer fresh perspectives on the molecular mechanisms that drive the malignancy of breast cancer and to identify possible therapeutic strategies to tackle this major public health issue.

## Materials and methods

### Cell culture

In this research, the MDA-MB-231 human breast cancer cell line was acquired from the Cell Bank at the Shanghai Institute of Cell Biology. These MDA-MB-231 cells were grown in Dulbecco’s Modified Eagle Medium (DMEM) (Beyotime, China), with an addition of 10% fetal bovine serum (FBS, Gibco, USA). All cells were kept in a humidified environment at 37℃ with 5% CO_2_, and the presence of mycoplasma was evaluated using Myco-Zero (Beyotime, China). The constructs sh-NC and sh-RANBP1 were produced by Nanjing Tsingke Biotech Co., Ltd., and the transfection was carried out with Lipofectamine 3000 Reagent (Thermo Fisher Scientific, China) over a period of 48 h. The lentiviral plasmids sh-NC or sh-RANBP1 were co-transfected alongside the packaging plasmid psPAX2 and pMD2.G. The efficiency of knockdown was confirmed through RT-qPCR or western blotting.

### RNA extraction and RT-qPCR

Cells were processed to extract total RNA with TRIzol reagent (Invitrogen, USA), The RNA’s concentration and purity were measured with a Nanodrop 2000 (Thermo Scientific, MA, USA). Reverse transcription of total RNA into cDNA was conducted using a cDNA reverse transcription kit (Takara, Japan). Quantitative RT-PCR was carried out employing SYBR Green PCR Master Mix (Invitrogen, USA) along with Applied Biosystems instruments. The relative expression levels were determined using the 2^-∆∆CT^ method. For information regarding the primer sequences utilized in quantitative PCR, please consult Table 1.

**Table 1:** Primers used in qRT-PCR

Sequence type	Sequence (5’–3’)
*RANBP1*	
Forward primer	GCTCGGGACGAGGATCAC
Reversed primer	CTCTCTCTTCGATCTCTTTCCTG
*MUC1*	
Forward primer	CCACGCCAGTGCTAAATTGG
Reversed primer	CCACGCCAGTGCTAAATTGG
*NR4A2*	
Forward primer	TTTGCCCTCGAAACCGAAGA
Reversed primer	GGGCACTGATCAGACTCACC
*Actin*	
Forward primer	CTATCACCTCCCCTGTGTG
Reversed primer	TCCCTTGCCCTCCTAAA
*RCC2*	
Forward primer	ACCAACACCTCCCGTGAATC
Reversed primer	AAGACTTGGGCTTGTGGTCC
*ZNF704*	
Forward primer	ACTCGCTCCATCTGTCTCCT
Reversed primer	CGGGAGGACTTCGAACCAAA
*SLCO4C1*	
Forward primer	GCCGTCACGCAAGGTATTGT
Reversed primer	GGCCAGTCAGGGAACTCTTC
*SCD*	
Forward primer	CTTGCGATATGCTGTGGTGC
Reversed primer	CCGGGGGCTAATGTTCTTGT
*WNT7B*	
Forward primer	GTGAAGCTCGGAGCACTGT
Reversed primer	GGCCAGGAATCTTGTTGCAG
*CTSB*	
Forward primer	CTGGGGTGACAATGGCTTCT
Reversed primer	GTACTGATCGGTGCGTGGAA
*ID1*	
Forward primer	AAACGTGCTGCTCTACGACA
Reversed primer	AAACGTGCTGCTCTACGACA
*F2RL2*	
Forward primer	AGGCGAGTCTCCTCATCCTT
Reversed primer	AGACTACCCAGGCACAAAGC
*LRPB*	
Forward primer	CCACCAGCCACAAGTGTGTA
Reversed primer	GACACTCGTTCAGCCAGGTA
*PRDM1*	
Forward primer	TCCAATCTGAAGGTCTGCCA
Reversed primer	CGCTTGTGCAGTTTCAGGTG

### Small RNA sequencing

Small RNA sequencing and analysis were performed by OE Biotech Co., Ltd. (Shanghai, China). Total RNA was extracted using TRIzol reagent according to the manufacturer’s protocol. RNA quantity and integrity were assessed using a Nanodrop 2000 spectrophotometer and an Agilent 2100 Bioanalyzer. For each sample, 1 µg of total RNA was used to construct a small RNA library using the NEBNext Small RNA Library Prep Set for Illumina (NEB, USA). Adapter-ligated RNAs were reverse transcribed into cDNA and amplified by PCR. The 140–160 bp PCR products were isolated as the final small RNA libraries. Library quality was confirmed, and sequencing was performed on the Illumina NovaSeq 6000 platform.Raw reads were first processed to remove low-quality reads, 5′ primer contaminants, and poly(A) tails. Clean reads were then aligned to reference databases including Rfam v10.1 and GenBank for non-coding RNA annotation. To identify known miRNAs, the reads were mapped to the miRBase v21 database. Unannotated reads were further analyzed using miRDeep2 to predict novel miRNAs based on hairpin structure features. Differential expression analysis of miRNAs was conducted using the DEG algorithm in R, applying thresholds of *p* < 0.05 and|log2 fold change| >1. GO and KEGG enrichment analyses of the predicted target genes of differentially expressed miRNAs were performed using R, based on the hypergeometric distribution.

### Western blot analysis

Cells were lysed on ice using RIPA lysis buffer, and the resulting supernatants were subjected to electrophoresis on a 10% SDS-PAGE gel. The proteins that were separated were then transferred onto a 0.45 μm polyvinylidene difluoride (PVDF) membrane (Millipore), followed by blocking with 5% skimmed milk and incubation with primary antibodies for 10 h at 4℃. The antibodies employed included anti-RANBP1 (diluted 1:1000, Proteintech, China), anti-Tubulin (1:10000 dilution, Proteintech, China), anti-WNT7B (1:500 dilution, Proteintech, China), and anti-CTSB (1:500 dilution, Proteintech, China). Subsequently, the membranes were incubated at room temperature for 1 h with labeled goat-anti-rabbit or goat-anti-mouse secondary antibodies (1:10000 dilution, Proteintech, China), and then stained using BeyoECL Plus (Beyotime, China).

###  Flow cytometry

The apoptosis detection was performed using the Annexin V-APC/7-AAD kit (Invitrogen, USA) via flow cytometry. Cells underwent treatment with the Annexin V binding solution and were stained with Annexin-V FITC and PI, followed by incubation. Subsequently, the samples were examined through flow cytometry (CytoFLEX, BECKMAN, CA, USA) utilizing FlowJo software for analysis.

### Statistical analysis

Statistical analyses were conducted using GraphPad Prism 7.0 (San Diego, CA, USA) along with R software (version 3.6.1). The data are expressed as mean ± standard deviation. A t-test was utilized to evaluate the differences between the two groups. For comparisons among multiple groups, one-way analysis of variance (ANOVA) was employed. A *p*-value of less than 0.05 was deemed significant.

## Results

### Differential analysis of MiRNA expression after RANBP1 knockdown

**Fig. 1:**
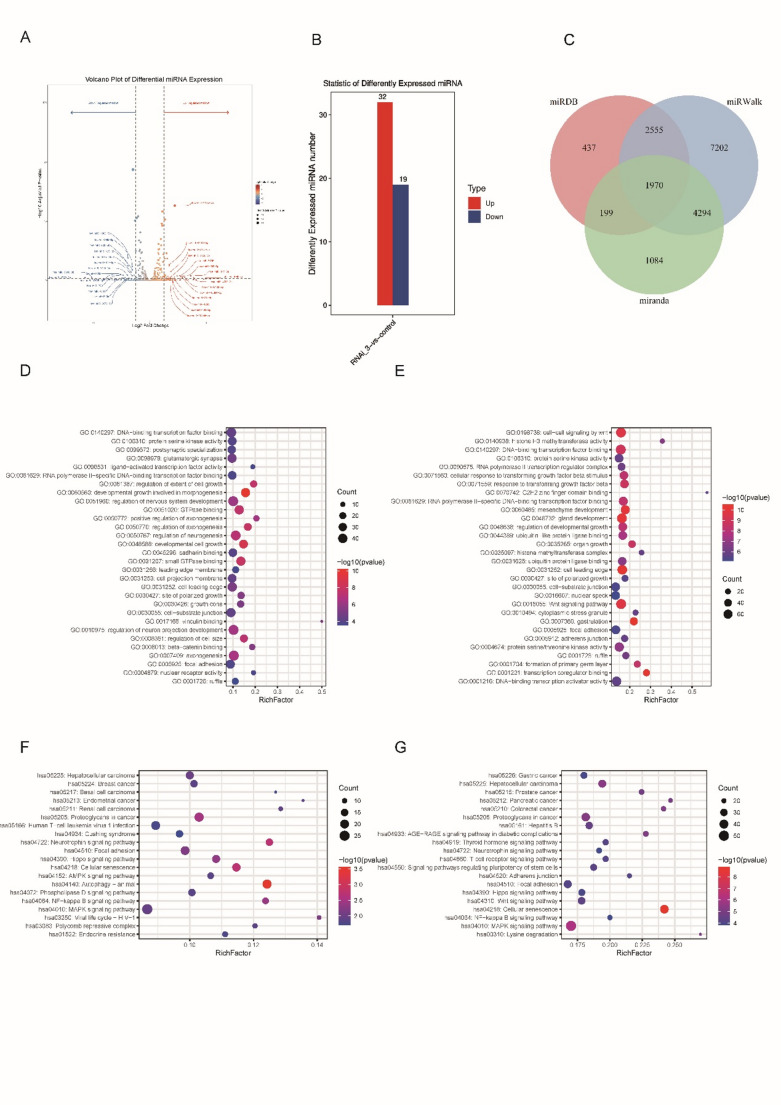
miRNA expression profiles and enrichment analysis. **A** Volcano plot of significantly differentially expressed miRNAs in two groups of MDA-MB-231 cells; **B** Histogram of significantly different miRNAs between two groups of MDA-MB-231 cells; **C** Venn plot of significantly different miRNAs using three software to predict target genes. **D** Go enrichment analysis results of significantly upregulated miRNA-targeted mRNAs (top 30); **E** Go enrichment analysis results of significantly down-regulated miRNA-targeted mRNA (top 30); **F** kegg enrichment of significantly up-regulated miRNA-targeted mRNA. analysis results (top 20); **G** kegg enrichment analysis results of significantly down-regulated miRNA-targeted mRNAs (top 20)

At the outset, we performed differential expression analysis of miRNA expression between the sh-NC and sh-RANBP1 groups within MDA-MB-231 cells (Fig. 1 A and B). Our analysis revealed a total of 51 miRNAs exhibiting notable expression differences, with 32 showing up-regulation and 19 showing down-regulation. Following this, we anticipated the target genes associated with these markedly differentially expressed miRNAs. To enhance the reliability of our predictions, we intersected the target genes identified by three distinct prediction algorithms, and used as our final target gene list. This approach yielded a final list of 1,970 target genes (Fig. 1C and Table S1).The results of the GO and KEGG enrichment analyses indicated that the target genes associated with up-regulated miRNAs were predominantly linked to pathways related to cancer, including the PD-1 and miRNA signaling pathways. In contrast, the target genes associated with down-regulated miRNAs were mainly engaged in the Wnt signaling pathway and various other pathways associated with tumor progression. (Fig. 1D–G).

### Differential analysis of mRNA profiles after RANBP1 knockdown

**Fig. 2:**
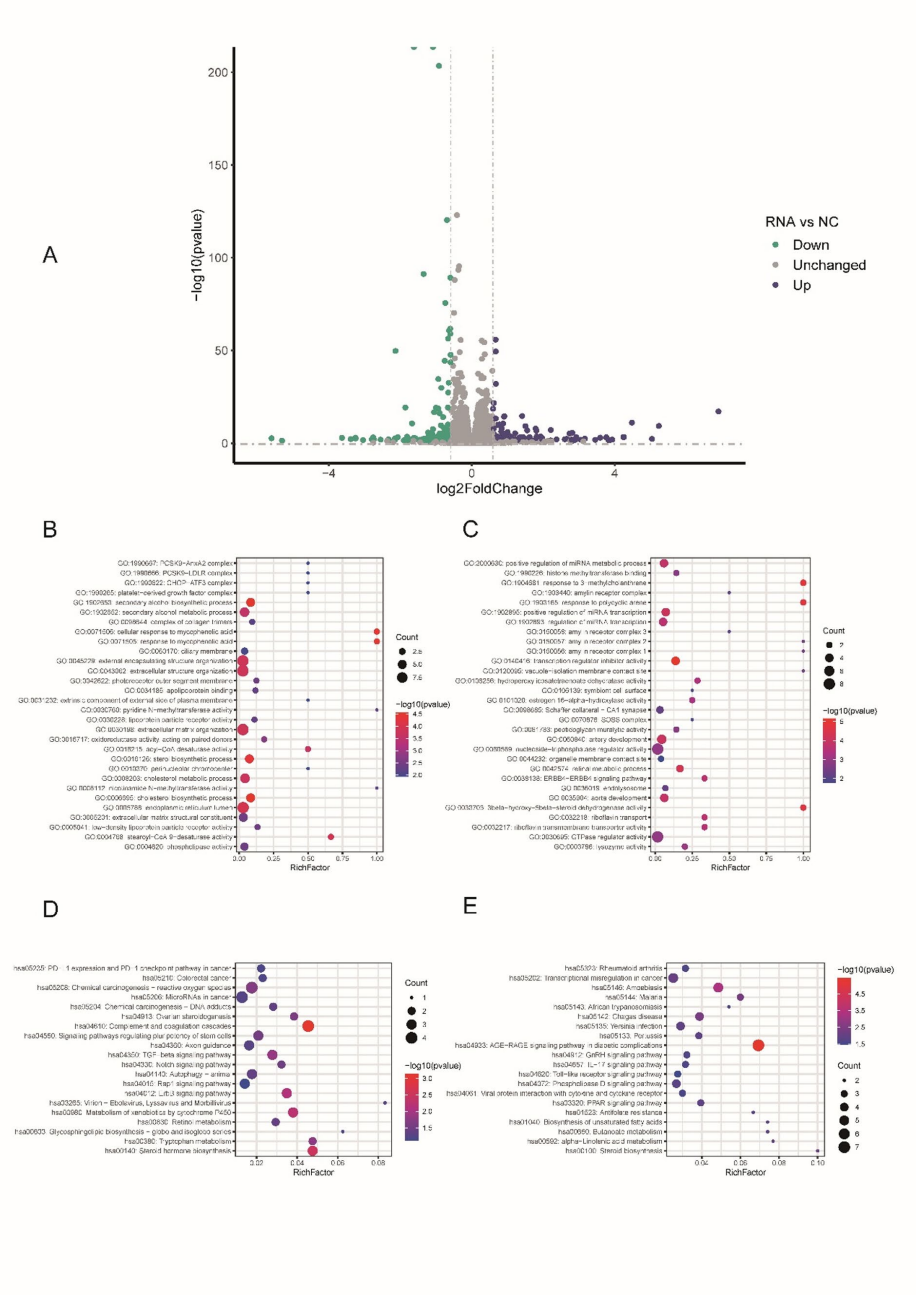
mRNA expression profiles and enrichment analysis. **A** Volcano plot of significantly different genes between the two transcriptomes of MDA-MB-231 cells; **B** Go enrichment analysis results of significantly upregulated mRNAs (top 30); **C** Go enrichment analysis results of significantly down-regulated mRNA (top 30); **D** KEGG enrichment of significantly up-regulated mRNA. analysis results (top 20); **E** KEGG enrichment analysis results of significantly down-regulated miRNA-targeted mRNAs (top 20)

To further investigate the changes in the cellular transcriptome following sh-RANBP1 treatment, we conducted transcriptome sequencing using two groups: sh-NC and sh-RANBP1, each comprising three replicates (Fig. 2A). Differential expression analysis identified a total of 342 significantly differentially expressed genes (DEGs), including 157 up-regulated and 185 down-regulated genes. Subsequently, we performed enrichment analysis using KEGG and GO for these DEGs (Fig. 2B–E). GO and KEGG enrichment analysis results showed that the up-regulated DEGs were primarily enriched in breast cancer and autophagy, while the down-regulated DEGs were mainly involved in tumor-related pathways such as Wnt and NF-kappa B signaling pathways.

### Paired miRNA- and mRNA analysis to identify novel miRNA-mRNA interactions

**Fig. 3:**
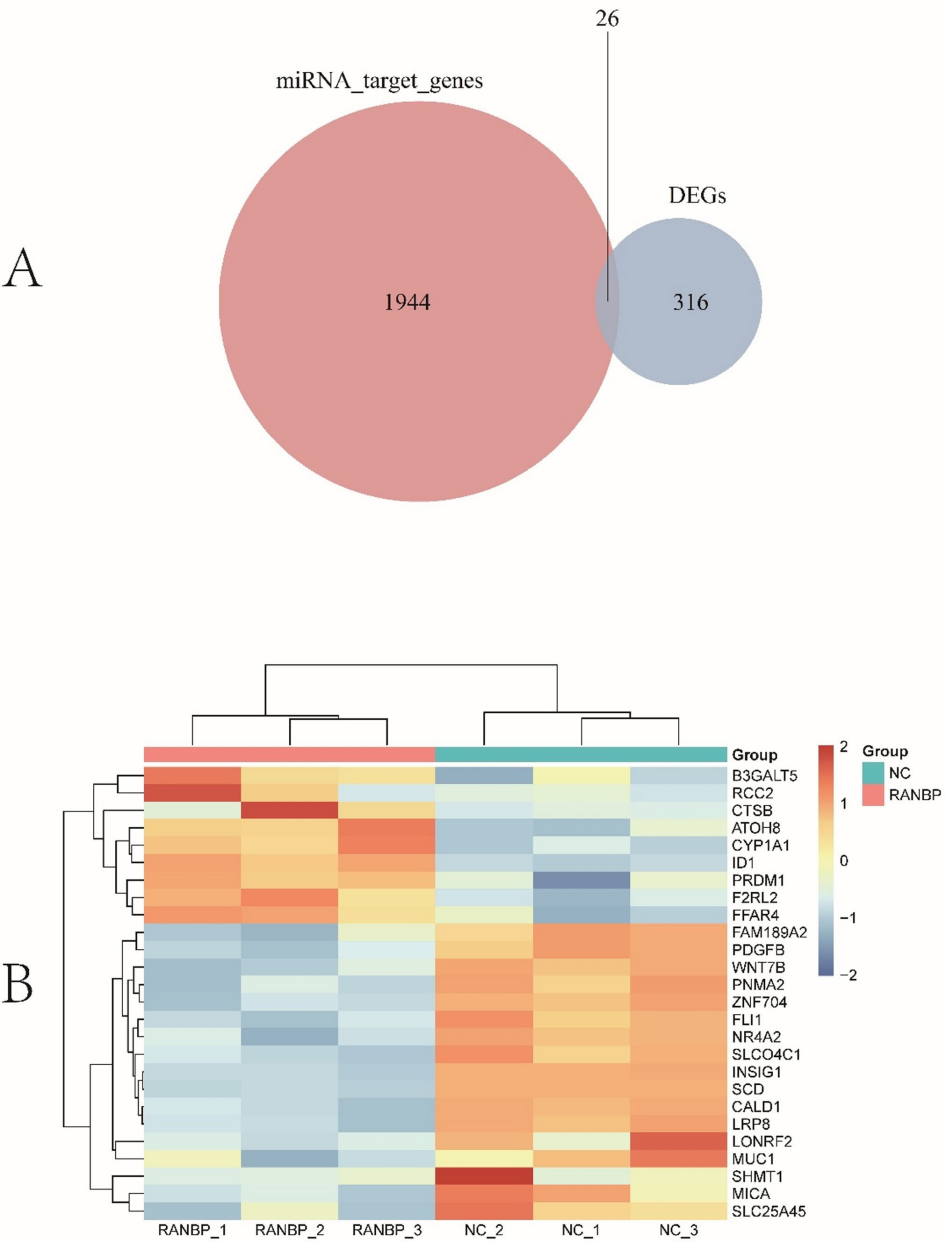
Paired analysis of miRNA and mRNA. **A** Venn diagram of target genes predicted by the software and significantly different mRNAs obtained by transcriptome sequencing; **B** Heat map of Log2 expression values, which have been z-score transformed for 26 genes identified in the Venn diagram

**Fig. 4:**
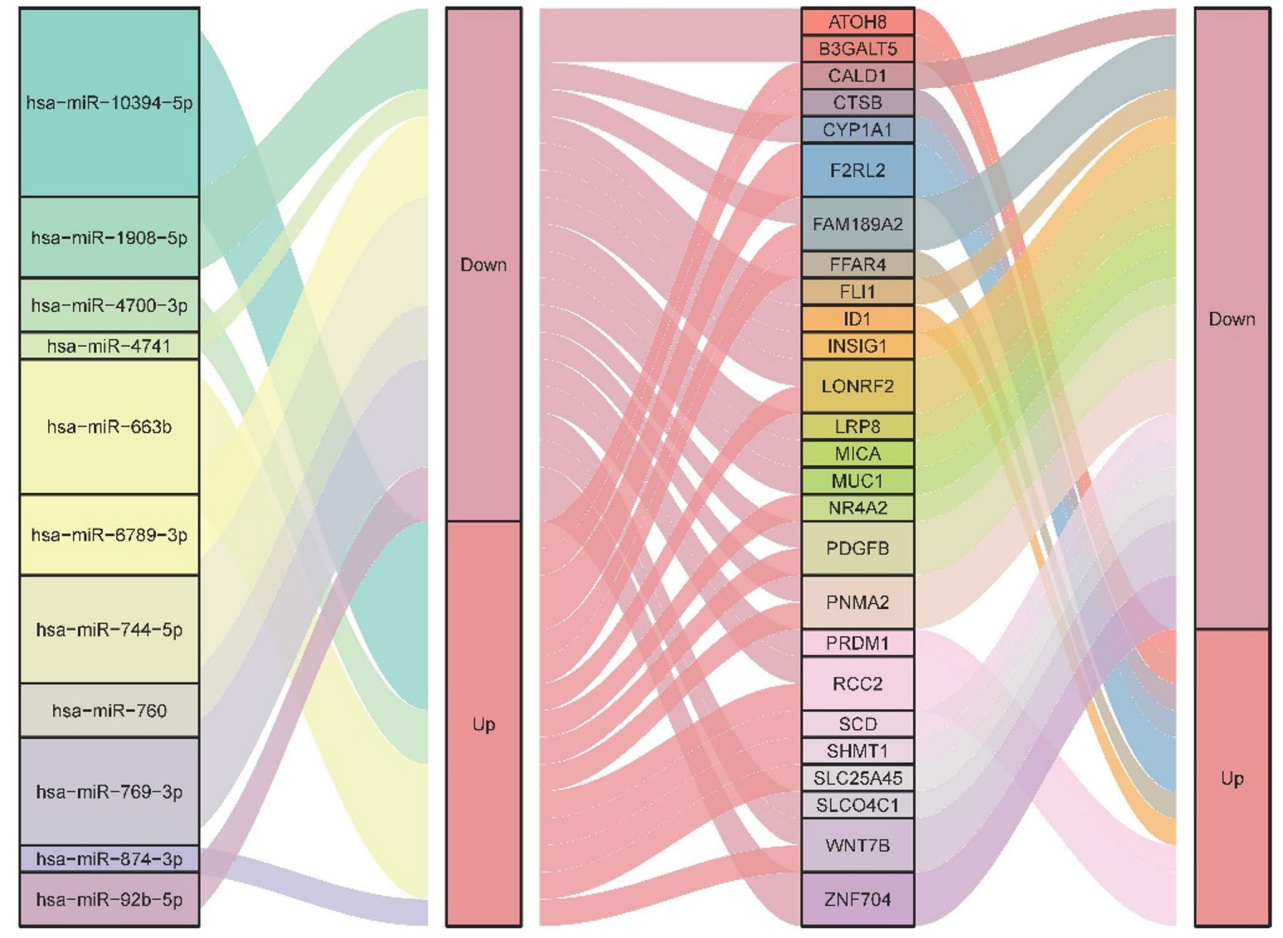
Sankey diagram of miRNA-mRNA regulatory network

To comprehensively assess the effects of sh-RANBP1 on breast cancer cells, we conducted a paired analysis of miRNA and mRNA. Initially, we identified the intersection of significantly DEGs between miRNA target genes and mRNA (Fig. 3A). This analysis yielded a set of 26 mRNAs that were significantly affected by differential miRNAs. Subsequently, we generated a heat map to visualize the expression patterns of these overlaped DEGs. The heat map revealed that genes such as *WNT7B* were notably down-regulated, while CTSB and *ID1* were significantly up-regulated in the sh-RANBP1 group (Fig. 3B). Notably, several of the genes were implicated in apoptosis-related pathways. The predicted miRNA-mRNA regulatory network is illustrated in Fig. 4. Based on the comprehensive analysis of miRNA and mRNA, we constructed a potential miRNA-mRNA regulatory network significantly influenced by sh-RANBP1 (Fig. 4). Our findings indicate that a larger proportion of mRNAs were ultimately downregulated.

### 3.4. qRT-PCR and WB to verify target gene expression

**Fig. 5:**
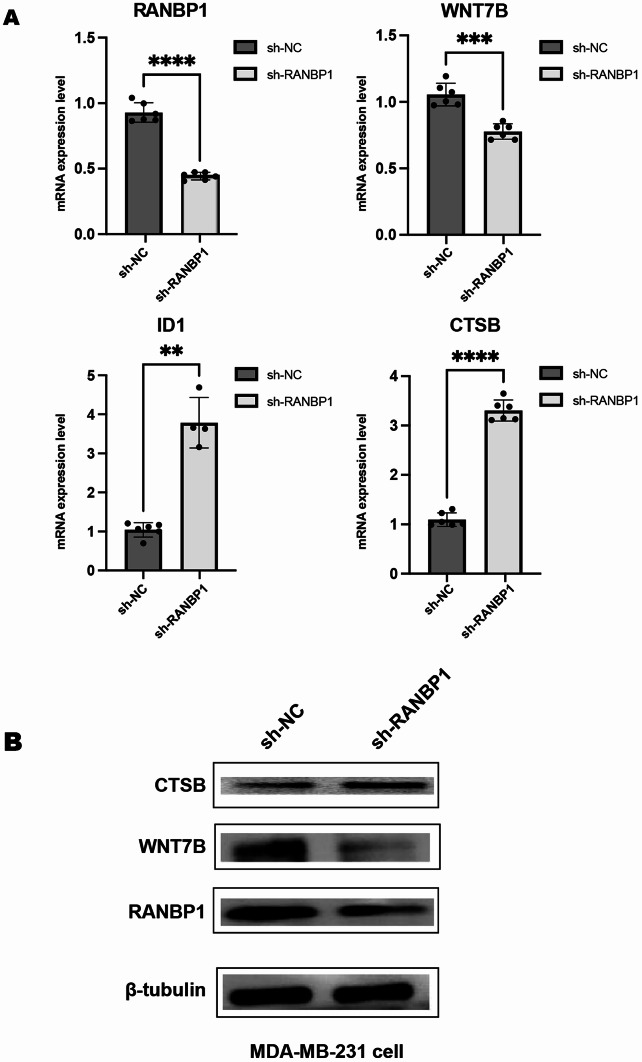
Validation of selected target gene expression after RANBP1 knockdown. **A** qRT-PCR analysis of mRNA expression levels of PANBP1, WNT7B, ID1, and CTSB in MDA-MB-231 cells transfected with sh-NC or sh-RANBP1. * *p* < 0.05, ** *p* < 0.01, *** *p* < 0.001, **** *p* < 0.0001. **B** Western blot analysis of CTSB, WNT7B, and RANBP1 protein expression in MDA-MB-231 cells following RANBP1 knockdown. β-tubulin was used as a loading control

To further validate our conclusions drawn from the transcriptome and miRNA sequencing analyses, we screened several genes for confirmation using qRT-PCR and WB (Fig. 5 and Figure S1). In the qRT-PCR results, we confirmed 13 genes, of which ten exhibited consistent trends with those shown in Figure S1. For instance, during the validation using qRT-PCR, notable differences were detected in the WNT7B, ID1, and CTSB genes between the two groups.Additionally, we verified the expression trends of proteins such as CTSB and WNT7B. The findings from the Western Blot analysis indicated a considerable rise in CTSB expression, which corresponds with the results illustrated in Fig. 5.

### Effect of sh-RANBP1 on breast cancer cell apoptosis

**Fig. 6:**
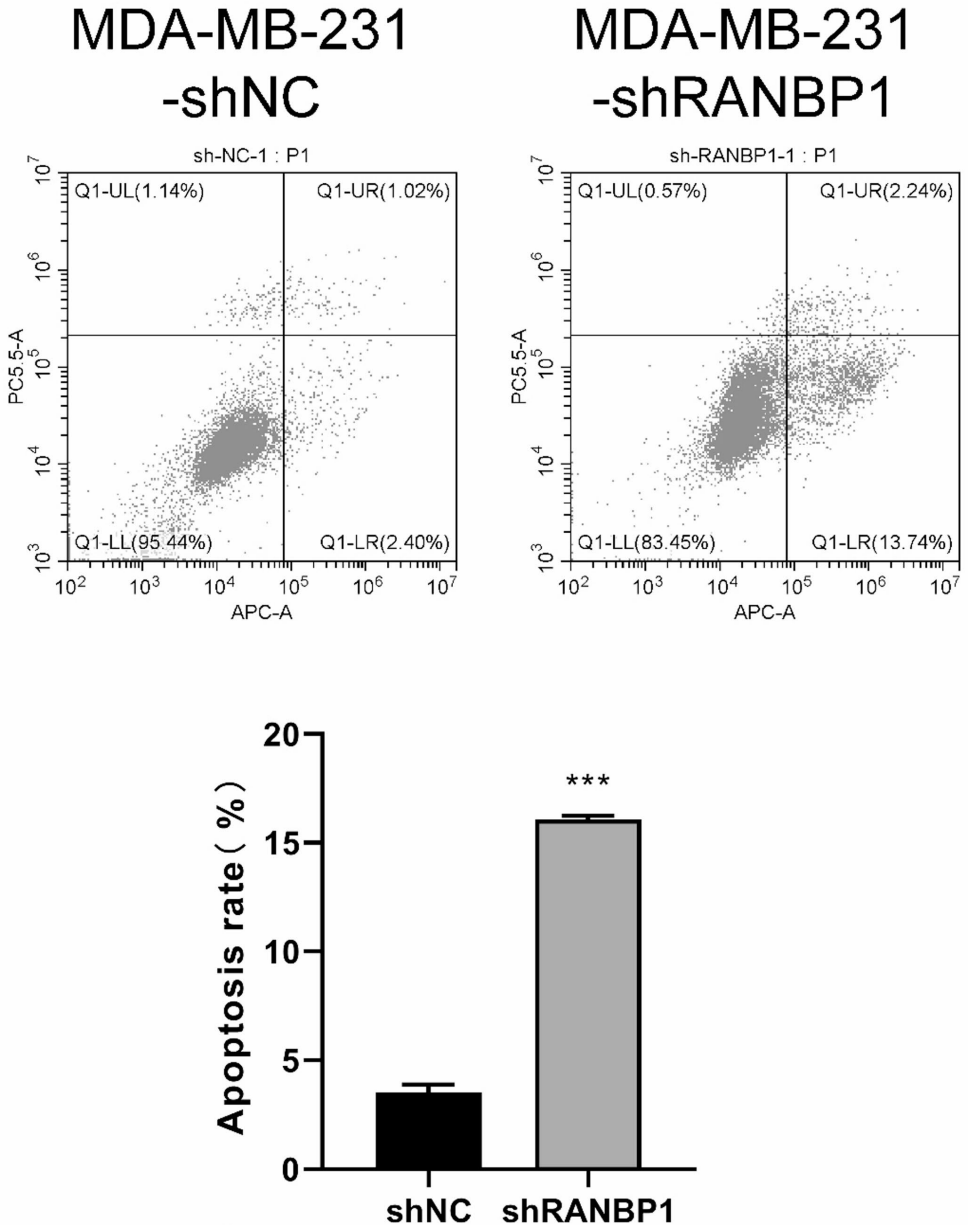
Flow cytometry to detect the difference in apoptosis of MDA-MB-231 cells between the two groups. Flow cytometry was used to detect the difference in apoptosis of MDA-MB-231 cells between the two groups; **A** Flow cytometry original result chart; **B** Flow cytometry statistical histogram

To evaluate the impact of sh-RANBP1 on apoptosis in breast cancer cells, we utilized flow cytometry to examine the rates of apoptosis after sh-RANBP1 treatment (Fig. 6). The findings revealed a notable rise in the percentage of apoptotic cells in the sh-RANBP1 treated group. These findings were concordant with the results of previous joint analyses of miRNA and mRNA.

## 4. Discussion

Breast cancer represents a major global health issue, as it is the most prevalent cancer type in women and one of the primary contributors to deaths associated with cancer (Wilkinson and Gathani 2022). In breast cancer, the evasion of apoptosis is a hallmark that facilitates tumor growth and resistance to therapies, making it a focal point for research and therapeutic strategies (Nagini 2017). The mechanisms underlying the evasion of apoptosis in breast cancer cells are complex and involve various genetic and epigenetic alterations (Bagacean et al. 2019). Modifications may result in an increased expression of anti-apoptotic proteins like Bcl-2, or a reduction in pro-apoptotic factors, thus shifting the equilibrium towards enhanced cell survival (Bagacean et al. 2019). For instance, the expression of miRNAs has been shown to play a crucial role in regulating apoptosis-related genes (Jovanovic and Hengartner 2006). Specific miRNAs, such as miR-29a, have been implicated in the modulation of apoptosis in breast cancer cells by targeting key regulators like Bcl-2 and p53, thereby influencing cell proliferation and survival (Grassilli et al., 2022). Recent research has underscored the promise of focusing on apoptotic pathways as a treatment approach for breast cancer (Yan et al., 2015; Baig et al. 2016). For example, the use of BH3 mimetics, which inhibit anti-apoptotic proteins, has shown promise in restoring sensitivity to apoptosis in resistant breast cancer cells (Zhang et al., 2007).Additionally, the combination of traditional chemotherapeutic drugs with apoptosis-enhancing agents has been explored to improve treatment outcomes (Qin et al., 2018). Moreover, the role of apoptosis in the tumor microenvironment is gaining attention (Yaacoub et al., 2016). High levels of apoptosis in tumors have been associated with increased infiltration of immune cells, which can enhance anti-tumor immunity (Zhu et al., 2019). This suggests that promoting apoptosis in breast cancer cells may not only directly kill cancer cells but also improve the overall immune response against the tumor (Jiang and Shapiro 2014).

RANBP1 has been identified as a significant factor in the context of various tumors, particularly in relation to CSCs and tumor progression (Kahm et al., 2023). Research indicates that RANBP1 is involved in the regulation of lung CSCs and GSCs, suggesting its role in cancer malignancy. Specifically, high expression levels of RANBP1 have been associated with a decreased survival rate in cancer patients, indicating its potential as a therapeutic target and a novel marker for cancer prognosis (Kahm et al., 2023). The mechanism by which RANBP1 influences tumor behavior includes its involvement in the regulation of miRNAs, which are crucial in the control of CSCs (Zheng et al., 2022). The findings of this research indicated that RANBP1-regulated miRNAs could influence the severity of cancers, highlighting the significant role of RANBP1 in the biology of tumors. Additionally, the expression of RANBP1 has been linked to the epithelial-mesenchymal transition (EMT), a process critical for cancer metastasis. In breast cancer, RANBP1 has been shown to be overexpressed, correlating with poor prognosis and aggressive tumor characteristics (Wei et al., 2021). The levels of RANBP1 in breast cancer tissues were notably greater than those in nearby normal tissues, and its increased expression correlated with several clinicopathological characteristics, such as the histological grade of the tumor and the occurrence of metastasis (Sheng et al., 2018). These findings suggested that RANBP1, along with its associated pathways, could serve as a valuable prognostic marker and therapeutic target in breast cancer management.

In breast cancer, miRNAs can influence various cellular processes such as proliferation, migration, invasion, and apoptosis (Tang et al., 2012). For instance, certain miRNAs are classified as oncomirs, which are upregulated in cancerous tissues and promote tumor initiation and progression (Cho 2007). Conversely, tumor suppressor miRNAs are typically downregulated in cancer, leading to the derepression of oncogenes and contributing to tumorigenesis (Rupaimoole et al., 2016). Research has shown that miRNAs are implicated in the modulation of key signaling pathways associated with breast cancer, including the Wnt/β-catenin and PI3K/AKT pathways (Rahmani et al., 2020). Aberrant activation of these pathways is often linked to tumor progression and poor prognosis in patients with breast cancer (Feng et al., 2018). For example, miRNAs such as miR-1267 and miR-2276 have been identified as potential tumor suppressors, with their downregulation correlating with higher grades of malignancy and advanced stages of breast cancer (Torkashvand et al., 2016). In this study, we observed that the sh-RANBP1 group upregulated the expression of several miRNAs, resulting in the upregulation of targeted regulatory genes such as *CTSB*, as well as other genes involved in apoptosis-related pathways. Conversely, CTSB was significantly upregulated following RANBP1 knockdown. CTSB (Cathepsin B) is a lysosomal protease that plays a dual role in cancer. While often associated with tumor invasion and metastasis, CTSB has also been reported to promote apoptosis through lysosomal membrane permeabilization and caspase activation under specific conditions (Droga-Mazovec et al. 2008; Aggarwal and Sloane 2014; Kågedal et al., 2001). The increased expression of CTSB observed in our study suggests that RANBP1 knockdown may sensitize breast cancer cells to apoptosis via lysosomal or mitochondrial apoptotic pathways. These findings further underscored the relationship between miRNA downregulation and the evasion of apoptosis in breast cancer cells. Moreover, the upregulation of apoptosis-related genes such as CTSB supports the hypothesis that miRNA–mRNA regulatory networks modulated by RANBP1 may contribute to cell death mechanisms (Gocheva et al. 2006). While we have identified potential regulatory networks, further experimental validation is essential. For example, the interaction between miRNA and mRNA necessitates additional verification through dual-luciferase assays. Confirming these regulatory networks will enhance our understanding of the interplay between breast cancer cells and apoptosis and may open new avenues for breast cancer treatment. Nevertheless, further in-depth experimental validation is necessary.

In summary, this study compared the expression profiles of miRNA and mRNA between the sh-RANBP1 group and the control group. Our findings indicated that sh-RANBP1 influenced miRNA expression, revealing significant differences based on the interaction network of miRNA and mRNA. The genes involved were primarily associated with apoptosis-related pathways. Subsequent WB and qRT-PCR results further validated our findings. Finally, flow cytometry confirmed an increased proportion of apoptosis in the sh-RANBP1 interference group, confirming the pro-apoptotic effects of RANBP1 knockdown.

## Supplementary Information

Below is the link to the electronic supplementary material.

Supplementary Material 1

Supplementary Material 2

Supplementary Material 3

## Data Availability

The initial dataset has been submitted to the NCBI repository (PRJNA1223670).
