# Supplementary figures and images for: The role of RANBP1 in regulating MiRNA expression and apoptosis in breast cancer cells
Source: Genes Genomics. 2025 Aug 21;47(10):1065–78. doi: 10.1007/s13258-025-01664-5 (PMC12515217; doi:10.1007/s13258-025-01664-5)

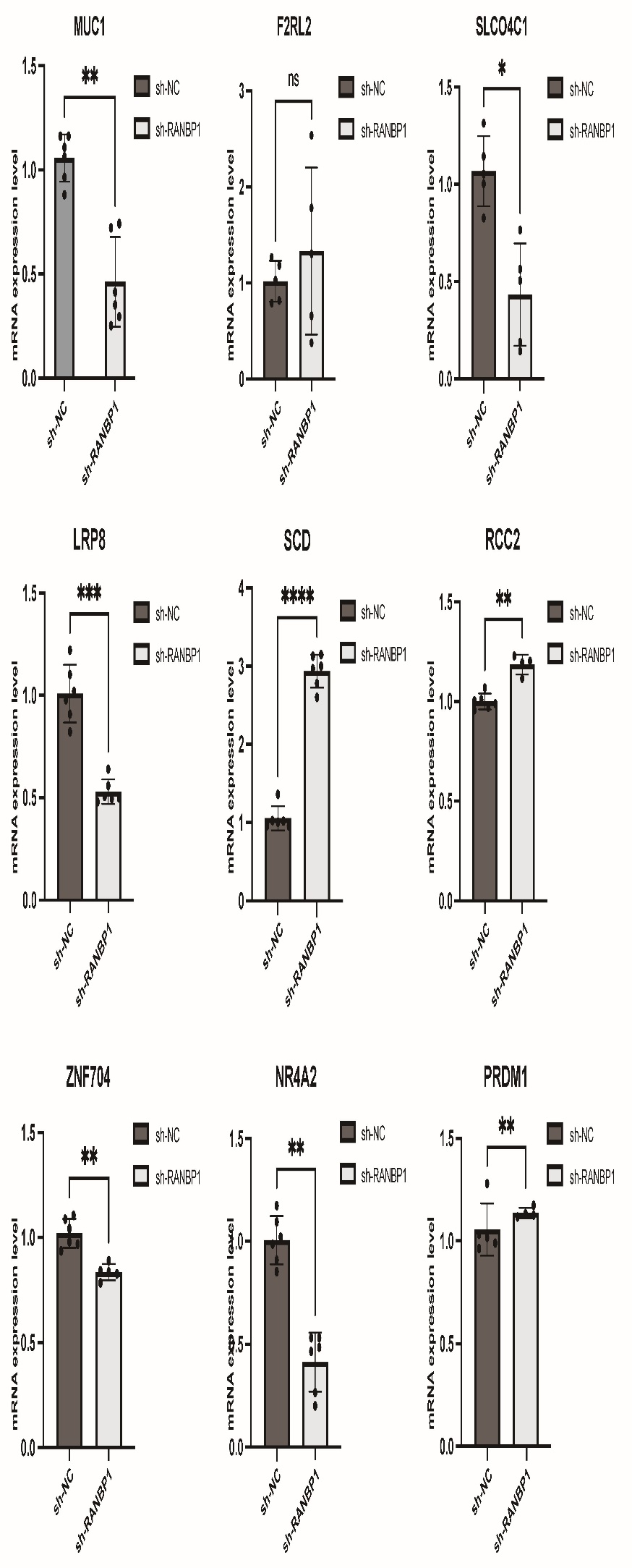


**Figure S1:** (A) q-RTPCR analysis detects the genes expression of two groups. * *p* < 0.05, ** *p* < 0.01, *** P < 0.001, **** P < 0.0001

Supplement: Supplementary file 1 — Supplementary Material 1 [file 13258_2025_1664_MOESM1_ESM.docx]
